# Matrix‐assisted autologous chondrocyte transplantation is effective at mid/long‐term for knee lesions: A systematic review and meta‐analysis

**DOI:** 10.1002/ksa.12549

**Published:** 2024-12-03

**Authors:** Alessandra Colombini, Vincenzo Raffo, Silvia Gianola, Greta Castellini, Giuseppe Filardo, Silvia Lopa, Matteo Moretti, Laura de Girolamo

**Affiliations:** ^1^ Orthopaedic Biotechnology Lab IRCCS Istituto Ortopedico Galeazzi Milan Italy; ^2^ Unit of Clinical Epidemiology IRCCS Istituto Ortopedico Galeazzi Milan Italy; ^3^ Faculty of Biomedical Sciences Università della Svizzera Italiana Lugano Switzerland; ^4^ Cell and Tissue Engineering Laboratory IRCCS Istituto Ortopedico Galeazzi Milan Italy; ^5^ Regenerative Medicine Technologies Laboratory, Laboratories for Translational Research (LRT) Ente Ospedaliero Cantonale (EOC) Bellinzona Switzerland; ^6^ Service of Orthopaedics and Traumatology, Department of Surgery EOC Lugano Switzerland; ^7^ Faculty of Biomedical Sciences, Euler Institute Università della Svizzera Italiana (USI) Lugano Switzerland

**Keywords:** chondral lesions, knee, matrix‐assisted autologous chondrocyte transplantation, membrane, meta‐analysis

## Abstract

**Purpose:**

This systematic review with meta‐analysis evaluates the long‐term efficacy of matrix‐assisted autologous chondrocyte transplantation (MACT) in terms of functional scores using scaffolds made of hyaluronic acid (HA) or collagen (C).

**Methods:**

Nineteen articles met the eligibility criteria for the analysis. Fourteen studies focused on patients treated with MACT with HA‐based scaffolds, four studies with C‐based scaffolds, and one study compared both scaffold types.

**Results:**

A higher percentage of patients in the HA subgroup had undergone previous cartilage repair procedures, whereas multiple lesions were more common in the C subgroup. Both HA‐ and C‐treated patients showed significant functional improvement in terms of International Knee Documentation Committee with overall mean differences at 2 and 5 years, and for HA‐treated patients at 10 years. Likewise, concerning the Tegner activity scale, both subgroups demonstrated significant improvement at 2 years, with the HA subgroup showing more sustained improvement up to 10 years. The HA subgroup also had EQ‐VAS reduction at 2, 5 and 10 years. Failure rates were similar between and within groups, with a range from 0% to 42% at different follow‐ups.

**Conclusion:**

Patients experienced mid‐term benefits from MACT, using both HA‐based and C‐based scaffolds, and long‐term benefits from using HA‐based scaffolds. The low failure rate and the fact that most patients did not require knee replacement surgery are encouraging. Accordingly, despite their complexity and high costs, regenerative techniques like MACT are effective, as they can significantly delay or even prevent the need for total knee replacement.

**Level of Evidence:**

Level IV.

**Abbreviations**
ACI: autologous chondrocyte implantation
BMI: body mass index
C: collagen
ES: effect size
HA: hyaluronic acid
ICRS: International Cartilage Repair Society
IKDC: International Knee Documentation Committee
MACT: matrix‐assisted autologous chondrocyte transplantation
MD: mean difference
MOOSE: meta‐analyses of observational studies in epidemiology
OB: outerbridge
OCD: osteochondritis dissecans

## INTRODUCTION

Cartilage defects of the knee affect the quality of life of patients and are considered a challenging clinical problem within the orthopaedic community. Because of the lack of vasculature and nerve network, cartilage lesions are characterized by a low spontaneous repair potential [3]. One approach to overcome this issue is to mimic the composition and properties of cartilage through the delivery of exogenous chondrocytes or tissue constructs grown in vitro. Autologous chondrocyte implantation (ACI), a regenerative cellular treatment for symptomatic cartilage defects, was first described by Brittberg et al. in 1994. Since then, it has evolved from first‐generation to fifth‐generation techniques [3]. First‐generation ACI involves the surgical injection of expanded autologous chondrocytes directly into the cartilage defect. This procedure requires the use of a harvested autologous periosteal flap, which needs extensive suturing and often leads to additional surgical interventions [15]. Matrix‐assisted autologous chondrocyte transplantation (MACT), a third‐generation ACI product, consists of chondrocytes delivered and cultured directly onto a membrane for surgical implantation and has been used for over 20 years with satisfactory results [26, 34]. MACT enhances chondrocyte delivery by seeding the chondrocytes onto a stable scaffold tailored to the size and shape of the cartilage defect, simplifies the surgical procedure, and reduces the length and number of incisions, thereby reducing overall surgical morbidity [3, 22, 33, 41].

The most commonly used scaffolds for MACT are based on hyaluronic acid (HA) and collagen (C), both representing cartilage structural components. They are biocompatible and provide excellent physical support for cells and for their extracellular matrix deposition [4, 16, 21, 22]. In a systematic review of this topic, a better impact on failures and revision surgeries has been reported when HA membranes were used in comparison with C scaffolds at mid‐term follow‐up [35]. However, many of the studies included in this systematic review did not allow for a meta‐analysis to be carried out and the clinical efficacy of MACT to be evaluated in the long term. As MACT is commonly indicated to address cartilage lesions in young patients, trying to avoid or at least postpone more invasive procedures, documenting long‐term results is of utmost importance to understand the real potential of this bioengineered approach. For this reason, the aim of the present systematic review with meta‐analysis is to assess up to a long‐term follow‐up the efficacy of MACT with various scaffold types to treat knee cartilage lesions.

## MATERIALS AND METHODS

The study was performed according to the Meta‐analyses Of Observational Studies in Epidemiology (MOOSE) guidelines to ensure the standard for reporting [43].

### Search strategy

A literature search was performed and launched on 31 March 2024 in PubMed, Scopus and the Cochrane Library databases for articles reporting the treatment with MACT of knee cartilage defects. The full search strategy is reported in Supporting Information S1: 1.

### Eligibility criteria

We included studies that fulfilled the following inclusion criteria: (a) use of MACT to treat knee cartilage defects; (b) assessment of International Knee Documentation Committee (IKDC) subjective score [20, 25, 45]; (c) minimum follow‐up of 24 months and (d) adults (age ≥18 years).

Articles published in languages other than English, or with no full‐text available, narrative reviews/meta‐analyses, preclinical studies (animal/in vitro studies), or studies out of scope (other joints, other pathologies, procedures other than MACT) were not included in the search.

### Description of study outcome(s)

The primary outcome was the clinical‐functional assessment by the subjective IKDC score (percentage ranging from 0 [severely disabled condition] to 100 [very good condition]) close to 2 years, 5 years, and 10 years. Furthermore, as secondary outcomes, we considered the function measured as Tegner scale (ranging from 0 [severely disabled condition] to 10 [very good condition]) [1, 2] and EQ‐VAS (ranging from 0 [worst imaginable health state] to 100 [best imaginable health state]) [30] close to 2 years, 5 years, and 10 years, and the overall failure rate.

### Study selection

After removing non‐English articles and articles with no full text available, a two‐step screening process was used to select eligible trials. First, titles and abstracts were screened by two independent reviewers (AC and VR) according to eligibility criteria. The eligible records were then systematically assessed for inclusion by reading full‐text articles. Any disagreement was solved by consensus between the two reviewers.

### Data extraction

Piloting a data collection form for the first studies and integrating important feedback from the reviewers, a standardized data collection form was used for data extraction by two other independent investigators (AC and VR).

The extracted data included characteristics of the studies (author, year of publication and geographic location of the corresponding author), patients demographic information (gender, age, number of patients and body mass index [BMI], percentage of previous and concomitant surgical procedures), injury features (defect location, defect aetiology, lesion size, type of lesion: isolated, multiple and kissing, lesion grade according to either outerbridge [OB] or International Cartilage Repair Society [ICRS]), type of previous and concomitant surgical procedures (ligament reconstruction, osteotomies, microfractures, lateral release, realignment procedures, meniscectomies, cartilage repair procedures, loose body, synovial and Hoffa removals, hardware removals, fracture fixation, tendon suture, patellar plastic, duplication of the medial retinaculum and patellar tendon scarification), composition of the scaffold (HA or C), chondrocytes transplantation protocol, length of follow‐up, outcomes (IKDC, TEGNER, EQ‐VAS, failure rate). For the quantitative synthesis of IKDC, TEGNER and EQ‐VAS, outcome data were grouped at 2 years, from 3 to 5 years, and over 5 years. We used the change values in the mean with their standard deviation.

### Quality assessment

Risk of bias was assessed using the NewCastle Ottawa scale adapted for observational studies with a single cohort (Supporting Information S1: 1) by two independent reviewers (AC and SL). Any disagreement was resolved by discussion.

### Statistical analysis

According to the distribution of data, descriptive statistics are presented as range, median (10–90 percentile), or absolute frequency (percentage), when appropriate. When possible, a meta‐analysis with a pooled mean difference (MD) of pre/post‐treatment effects and 95% confidence intervals (CIs) was performed for primary and secondary continuous outcomes subgrouping data according to treatments (HA and C subgroups). A random study effects model with at least two studies was applied. Heterogeneity was evaluated using the *I* ^2^ statistic [7], where *I* ^2^ > 75% was considered substantial heterogeneity. Therefore, in the presence of substantial heterogeneity, attempts have been made to explain clinical or methodological heterogeneity using subgroup or sensitive analysis. For failure rate, we visually inspected the data with a funnel plot, built with the metaprop command in STATA software 16 for proportional meta‐analyses. The effect size (ES) represented the percentage of failures grouped by type of scaffold between the values of 0 and 1. The forest plot presented proportions with 95% exact CIs for each study.

Data analyses were performed using RevMan Software 5.4 [44]. For hypothesis testing, a probability value of <0.05 was considered statistically significant. All statistical tests were two‐sided.

## RESULTS

### Study selection and characteristics of the studies

**Figure 1:**
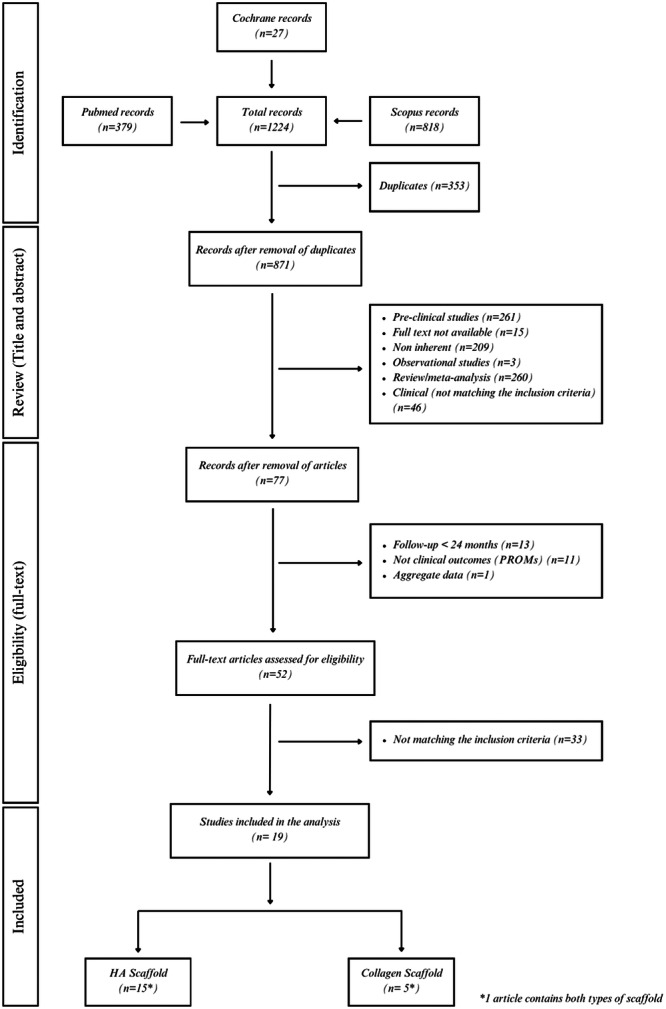
Flowchart of publications throughout the review process.

The search query retrieved 27 studies in Cochrane, 379 studies in PubMed and 818 studies in Scopus. After excluding 353 duplicates, the remaining 871 studies were selected based on their titles matching inclusion and exclusion criteria, resulting in 77 studies. Other articles were excluded after reviewing the abstract, reaching a selection of 52 articles that were further assessed according to the eligibility criteria. Of these, 33 did not match the inclusion criteria after reading the full text. At the end of the process, 19 articles that satisfied the eligibility criteria were selected for the analysis (Figure 1).

**Figure 2:**
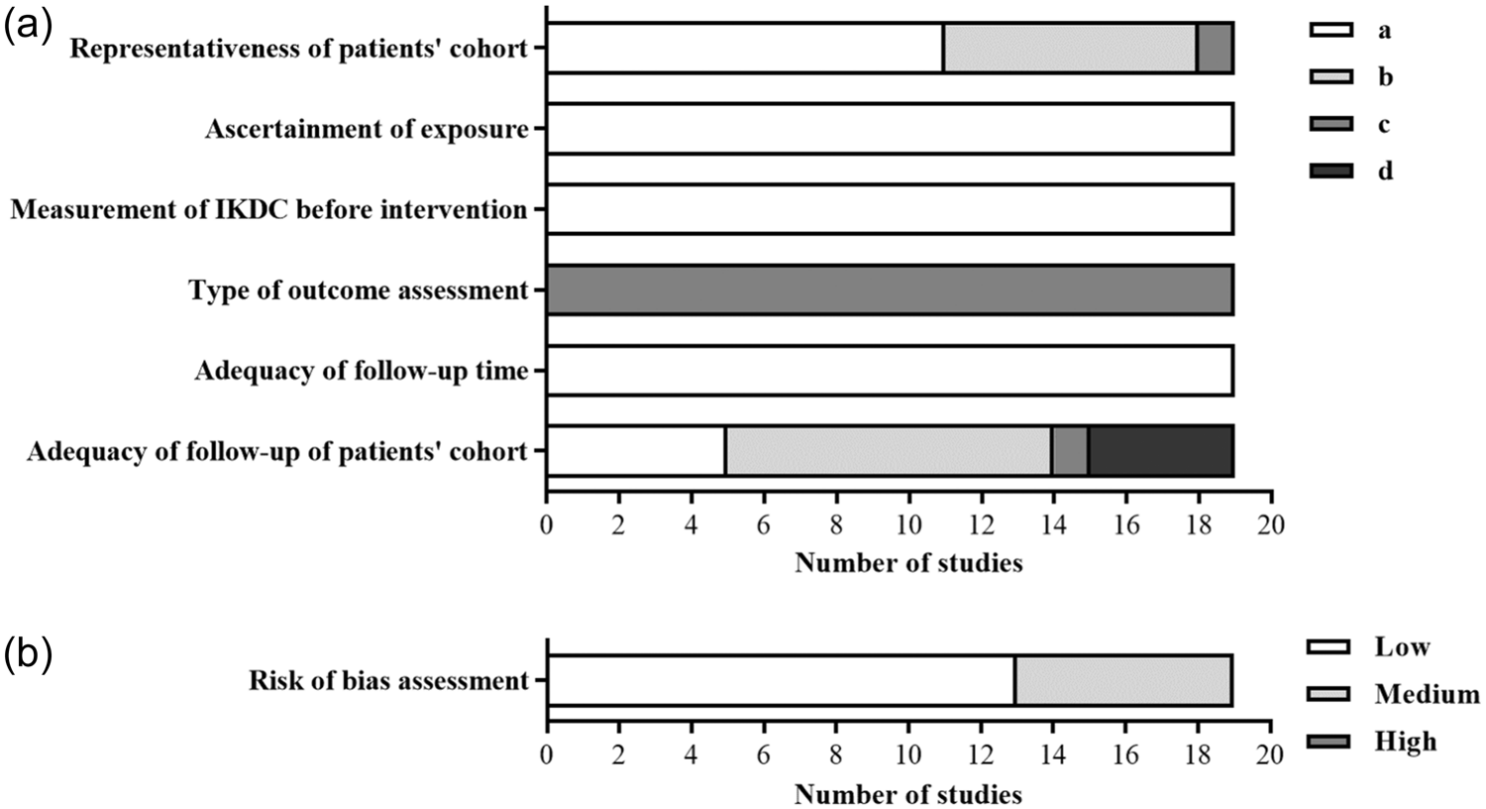
Risk of bias assessment. Score for each item within the selection and outcome categories (a). Study classification based on risk of bias (b). IKDC, International Knee Documentation Committee.

Overall, 14 papers described patients treated with MACT and HA‐based scaffolds, 4 studies described patients treated with MACT and C‐based scaffolds and 1 article reported the results of both scaffold types. Table 1 reports all the studies included in the qualitative and quantitative analysis. All the papers were published between 2005 and 2021. The studies conducted with HA‐based scaffolds were mainly conducted in Italy, of which many were from the same research group. Figure 2 shows that 68.4% of studies are at low and 31.6% of studies are at medium risk of bias.

**Table 1:** Study characteristics.

Years	Authors	Study type	Number of patients	Mean age of patients	Outcomes	Follow‐up (years)	Ref.	
**HA subgroup**								
2005	Marcacci et al.	Retrospective cohort study	192	37.6	IKDC	3.3	[31]	
2006	Nehrer et al.	Case series	36	33.0	IKDC	2; 3	[37]	
2009	Kon et al.	Cohort study	40	29.0	IKDC, EQ‐VAS	5	[27]	
2009	Gobbi et al.	Case series	34	31.2	IKDC, EQ‐VAS	2; 5	[18]	
2011	Filardo et al.	Case series	62	28.1	IKDC, Tegner, EQ‐VAS	2; 7	[12]	
2011	Kon et al.^a^	Case series	22	46.1	IKDC	2; 5	[28]	
2012	Filardo et al.	Case series	54	34.7	IKDC, Tegner, EQ‐VAS	2; 6	[13]	
2013	Filardo et al.	Case series	44	42.0	IKDC, Tegner, EQ‐VAS	2; 5; 9	[14]	
2013	Filardo et al.	Cohort study	56 F; 56 M	34.0 F; 33.2 M	IKDC, Tegner, EQ‐VAS	2; 5	[11]	
2014	Filardo et al.	Cohort study	49	31.5	IKDC, Tegner, EQ‐VAS	2; 5	[9]	
2014	Filardo et al.	Case series	131	29.2	IKDC, Tegner, EQ‐VAS	2; 5; 7	[10]	
2015	Gobbi et al.	Nonrandomized prospective trial	19	43.1	IKDC, Tegner	>3	[17]	
2016	Kon et al.	Case series	32	31.3	IKDC, Tegner, EQ‐VAS	2; 5; 10	[29]	
2019	Zaffagnini et al.	Case series	31	22.6	IKDC, Tegner, EQ‐VAS	2; 10	[47]	
2020	Zaffagnini et al.	Case series	23	29.1	IKDC, Tegner	12	[46]	
**C subgroup**								
2010	Niemeyer et al.	Case series	59	37.0	IKDC, Tegner	2	[38]	
2011	Kon et al. a	Case series	39	45.2	IKDC	2; 5	[28]	
2012	Marlovits et al.	Case series	21	35.2	IKDC	5	[32]	
2014	Ibarra et al.	Case series	10	35.0	IKDC, Tegner	2; 3	[24]	
2021	Zak et al.	Case series	21	31.0	IKDC, Tegner	2	[48]	

Abbreviations: C, collagen; HA, hyaluronic acid; IKDC, International Knee Documentation Committee.

^a^ Same study.

### Patient features

The demographic features of the patients and the main clinical information are reported in Table 2.

**Table 2:** Characteristics of the patients of the studies included in the meta‐analysis.

			Gender			
	Age (range)	BMI (range)	Female (%)	Male (%)	Previous treatments (%)	Concomitant treatments (%)
HA subgroup	22–46	24–25	32	68	65	44
C subgroup	31–45	24–26	33	67	31	39

*Note*: Eight missing values for ‘BMI’; three missing values for ‘Previous treatments’; four missing values for ‘Concomitant treatments’.

Abbreviations: BMI, body mass index; C, collagen; HA, hyaluronic acid.

Overall, 1031 records were included in the analysis, 881 of which were in the HA subgroup and 150 in the C subgroup. The two subgroups were similar in BMI and sex distribution, whereas differences were observed in the percentage of previous and concomitant treatments, with patients in the HA subgroup having undergone more previous treatments than those in the C subgroup.

### Cartilage defect features

The characteristics of the lesions treated with MACT are reported in Table 3. In both subgroups, the defects were mainly located at the femoral level, followed by the patella, trochlea and tibia. On the contrary, 16.7% of multiple lesions were present in the C subgroup, whereas 6.6% were present in the HA subgroup. Most of the lesions were of degenerative aetiology in both subgroups, followed by traumatic and associated osteochondritis dissecans (OCD). HA showed 12.1% of OCD in comparison with the 6.7% of the C subgroup. In all the studies, the lesions were of Grade III/IV ICRS or IV OB.

**Table 3:** Characteristics of the cartilage defects.

	Defect location (%)					Defect aetiology (%)			
	Femur	Patella	Trochlea	Tibia	Multiple	Traumatic	Degenerative	OCD	Defect size (range, cm^2^)
HA subgroup	70.9	13.4	8.5	0.4	6.6	33.5	54.4	12.1	2.0–9.7
C subgroup	61.6	17.4	4.3	0.0	16.7	23.3	70.0	6.7	1.0–5.1

*Note*: One missing value for ‘OCD’ in HA subgroup; three missing values for ‘Defect aetiology’ in C subgroup.

Abbreviations: C, collagen; HA, hyaluronic acid; OCD, osteochondritis dissecans.

### Previous and concomitant surgical procedures

An accurate assessment of the patient's surgical history is crucial to plan a MACT and maximise the chances of success. Previous surgical procedures may compromise or favour the success of MACT through various mechanisms, mainly affecting tissue quality and joint stability. Table 4 reports the percentages of previous and concomitant surgical procedures in the two subgroups. The patients in the HA subgroup had previously undergone 65.0% of surgical procedures in comparison with the 30.1% of the patients in the C subgroup. In particular, the most performed previous procedures in the HA subgroup were cartilage repair (20.0%) and ligament reconstruction (17.9%), whereas in the C subgroup, ligament reconstruction (14.7%) and meniscectomies (10.0%). Noticeably, many more cartilage repair procedures were performed in the HA subgroup (10 times more than in the C subgroup). Conversely, ligament reconstruction and meniscectomies were performed in similar proportions in the two subgroups, as were osteotomy and lateral release.

**Table 4:** Previous and concomitant surgical procedures.

	Previous		Concomitant	
	HA subgroup (%)	C subgroup (%)	HA subgroup (%)	C subgroup (%)
Cartilage repair procedures	20.0	2.0	0.1	4.0
Ligament reconstruction	17.9	14.7	16.2	8.7
Meniscectomies	15.3	10.0	11.7	2.7
Osteotomies	0.3	0.7	6.6	14.7
Lateral release	1.7	1.3	2.2	2.0
Microfractures	3.0	0	0.3	1.3
Realignment procedures	2.2	0	4.2	2.7
Loose body, synovial and Hoffa removals	1.8	0.7	2.8	1.3
Other procedures	2.8	0.7	0.3	2.0

*Note*: One study [31] was excluded because different treatments, such as meniscus and/or ligament surgery and osteotomies, were grouped together. Other procedures: hardware removals, fracture fixation, tendon suture, patellar plastic, duplication of the medial retinaculum and patellar tendon scarification.

Abbreviations: C, collagen; HA, hyaluronic acid.

Concomitant surgical procedures were performed in 44.4% and 39.4% of patients in the HA and C subgroups, respectively. In particular, the most frequent concomitant procedures were ligament reconstruction (16.2%) and meniscectomies (11.7%) in the HA subgroup, and osteotomies (14.7%) and ligament reconstruction (8.7%) in the C subgroup. Comparing the two subgroups, 16.2% ligament reconstruction and 11.7% meniscectomies were performed in the HA subgroup in comparison with 8.7% and 2.7% in the C subgroup, respectively, whereas 14.7% osteotomies, 4.0% cartilage repair procedures and 1.3% microfractures were performed in the C subgroup in comparison with 6.6%, 0.1% and 0.3% in the HA subgroup, respectively.

### Clinical outcomes

#### International Knee Documentation Committee

Overall, 17 studies reported the outcome of interest at 2 years, among which 13 for the HA subgroup (818 records) and 4 for the C subgroup (129 records). Both subgroups showed a statistically significant functional improvement after treatment (overall: mean difference [MD] = −32.97, 95% CI = −36.18 to −29.76 [(*I* ^2^ = 80%]; HA subgroup: MD = −34.00, 95% CI = −37.62 to −30.38 [*I* ^2^ = 77%]; C subgroup: MD = −28.87, 95% CI = −30.84 to −26.91 [*I* ^2^ = 0%]).

Overall, 12 studies reported the outcome of interest at 5 years, 10 for the HA subgroup (537 records) and 2 for the C subgroup (60 records). Both subgroups showed a statistically significant functional improvement at 5 years (overall: MD = −33.06, 95% CI = −36.25 to −29.86 [*I* ^2^ = 59%]; HA subgroup: MD = −32.34, 95% CI = −35.72 to −28.95 [*I* ^2^ = 58%]; C subgroup: MD = −37.84, 95% CI = −49.68 to −26.00 [*I* ^2^ = 75%]).

**Figure 3:**
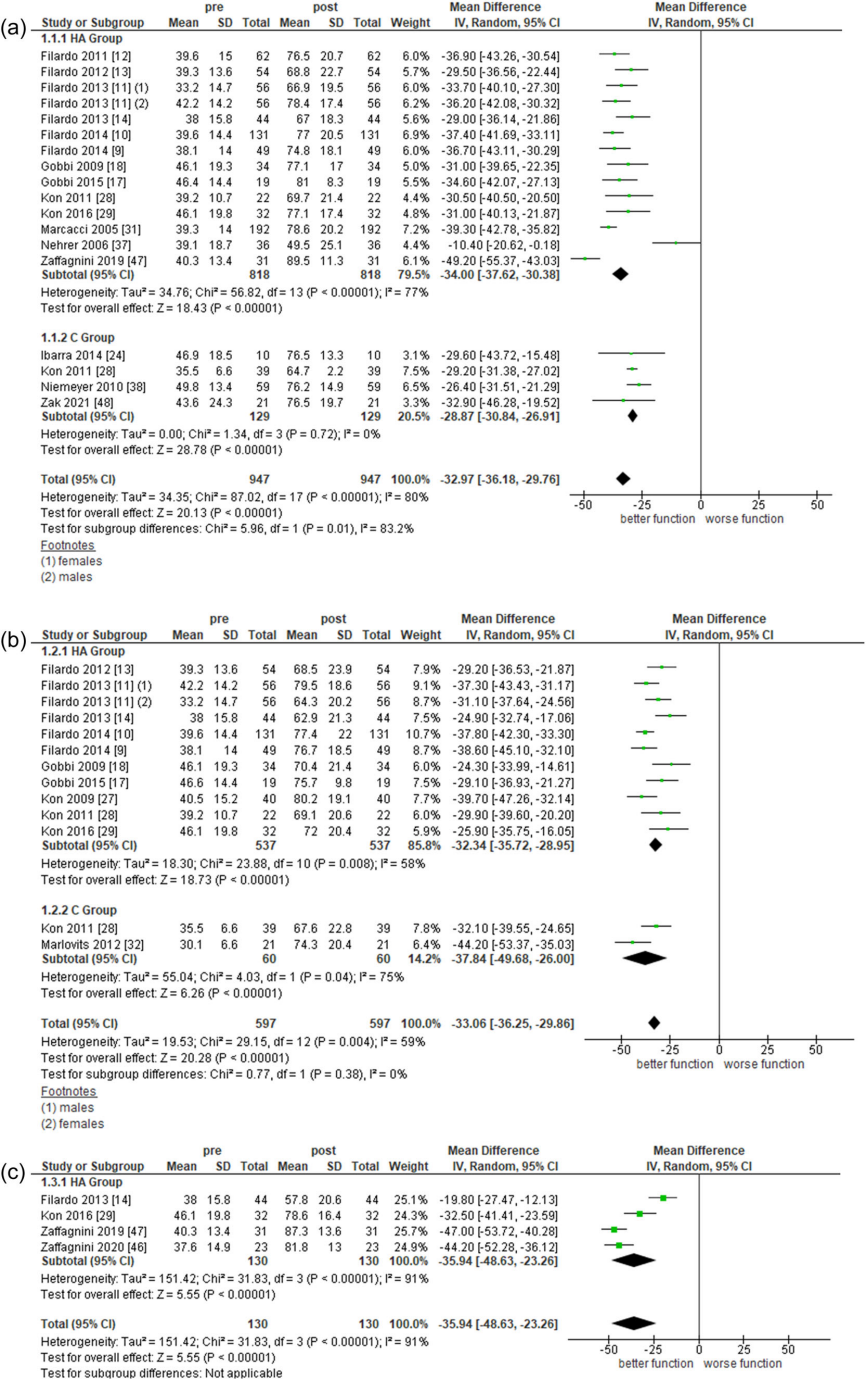
Meta‐analysis of pre‐post treatment on IKDC at 2 (a), 5 (b) and 10 (c) years. IKDC, International Knee Documentation Committee.

Four studies including 260 patients in the HA subgroup assessed IKDC at 10 years of follow‐up. Data showed a significant functional improvement of −35.94 points (95% CI = −48.63 to −23.26 [*I* ^2^ = 91%]). Full data analysis is reported in Figure 3.

#### Tegner

Six studies for the HA subgroup (369 records) and three for the C subgroup (90 records) reported outcomes measured with the Tegner Activity scale at 2 years. Both subgroups showed a statistically significant improvement in function after treatment (Overall: MD = −2.78, 95% CI = −3.57 to −1.99 [*I* ^2^ = 94%]; HA subgroup: MD = −3.04, 95% CI = −4.05 to −2.03 [*I* ^2^ = 95%]; C subgroup: MD = −1.86, 95% CI = −2.42 to −2.29 [*I* ^2^ = 33%]).

Five studies for the HA subgroup (256 records) reported the same functional score at 5 years, with a statistically significant improvement (MD = −2.53, 95% CI = −3.02 to −1.68 [*I* ^2^ = 86%]). Four studies for the HA subgroup (130 records) also presented Tegner scores at 10 years, again showing a statistically significant improvement (MD = −2.81, 95% CI = −4.47 to −1.15 [*I* ^2^ = 94%]).

**Figure 4:**
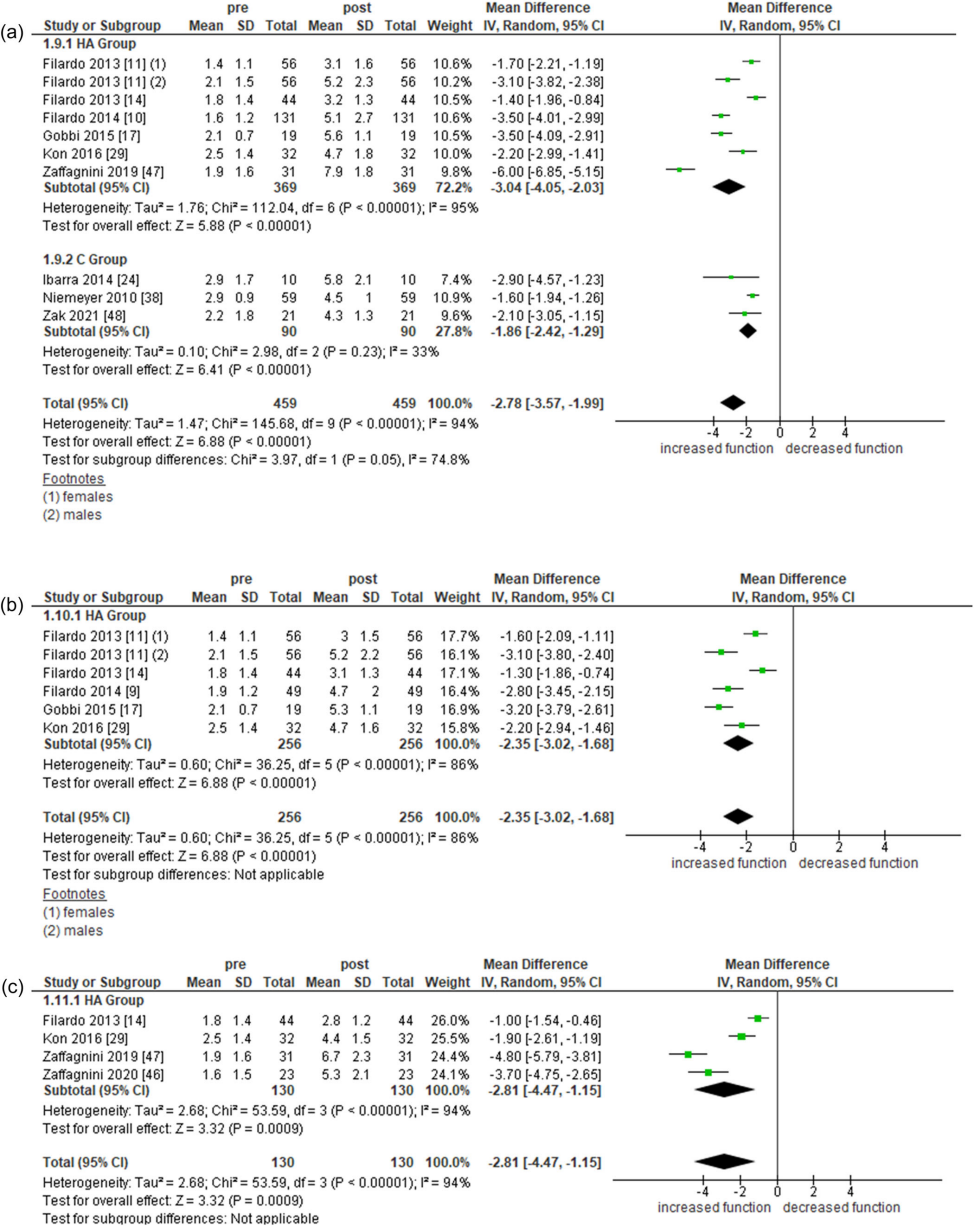
Meta‐analysis of pre‐post treatment on Tegner at 2 (a), 5 (b) and 10 (c) years.

Full data analysis is reported in Figure 4.

#### EQ‐VAS

Three studies assessing HA (110 records) measured EQ‐VAS at 2 years, showing a statistically significant reduction of pain (MD = −21.09; 95% CI = −28.23 to −13.94 [*I* ^2^ = 62%]). Four studies assessing HA (159 records) reported a statistically significant reduction of pain after treatment (MD = −20.68; 95% CI = −27.66 to −13.70 [*I* ^2^ = 68%]) in EQ‐VAS at 5 years. Likewise, three studies evaluating HA (107 records) measured EQ‐VAS at 10 years, again with an overall significant reduction of pain (MD = −21.9; 95% CI = −35.87 to −6.52 [*I* ^2^ = 92%]).

**Figure 5:**
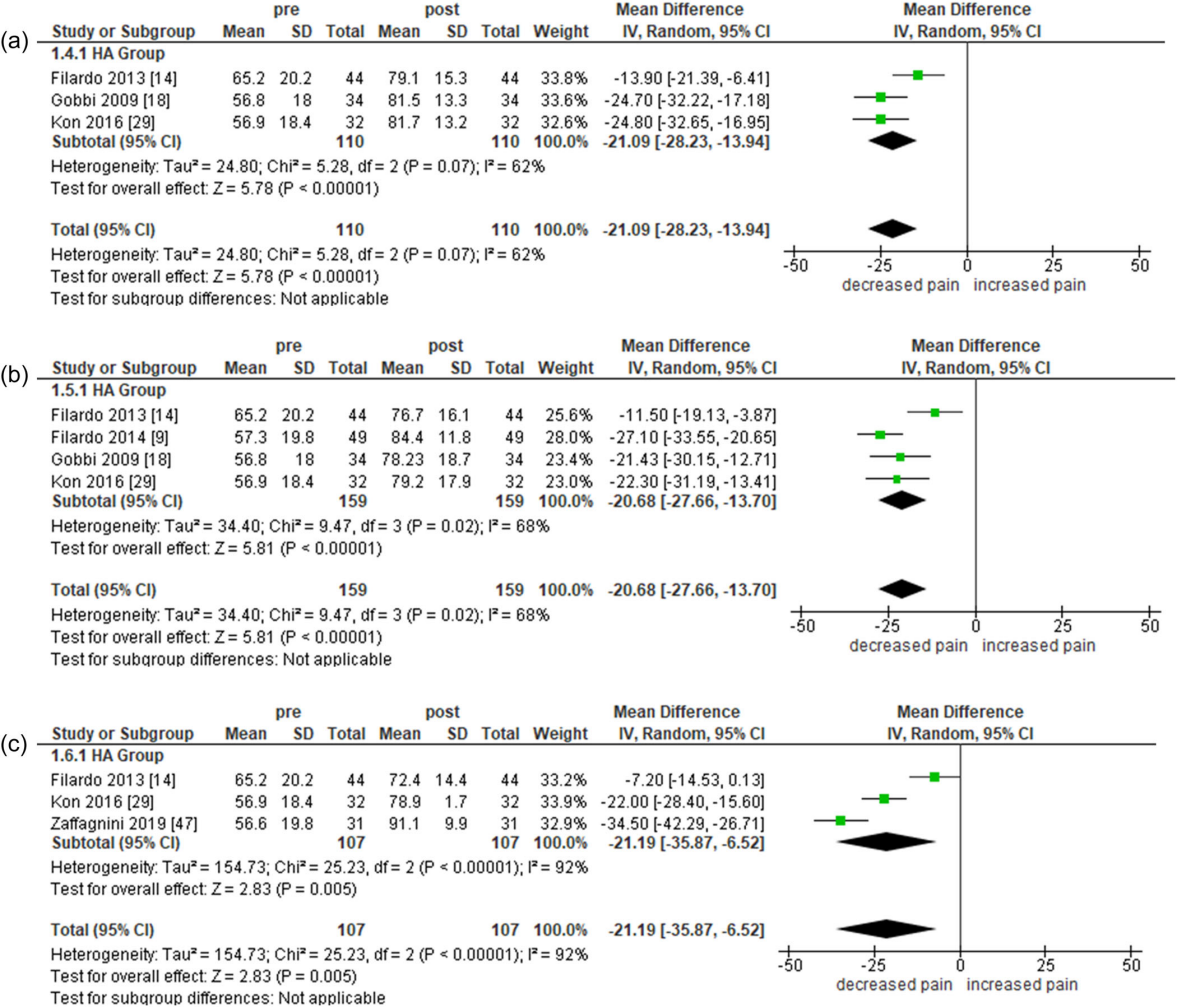
Meta‐analysis of pre‐post treatment on EQ‐VAS at 2 (a), 5 (b) and 10 (c) years.

Full data analysis is reported in Figure 5.

#### Failure rate

Sixteen studies reported failure rates, meaning the presence of pain and swelling [8] or graft failure, of which 12 studies were for the HA subgroup, 3 studies were for the C subgroup and 1 study reported data about both subgroups.

**Figure 6:**
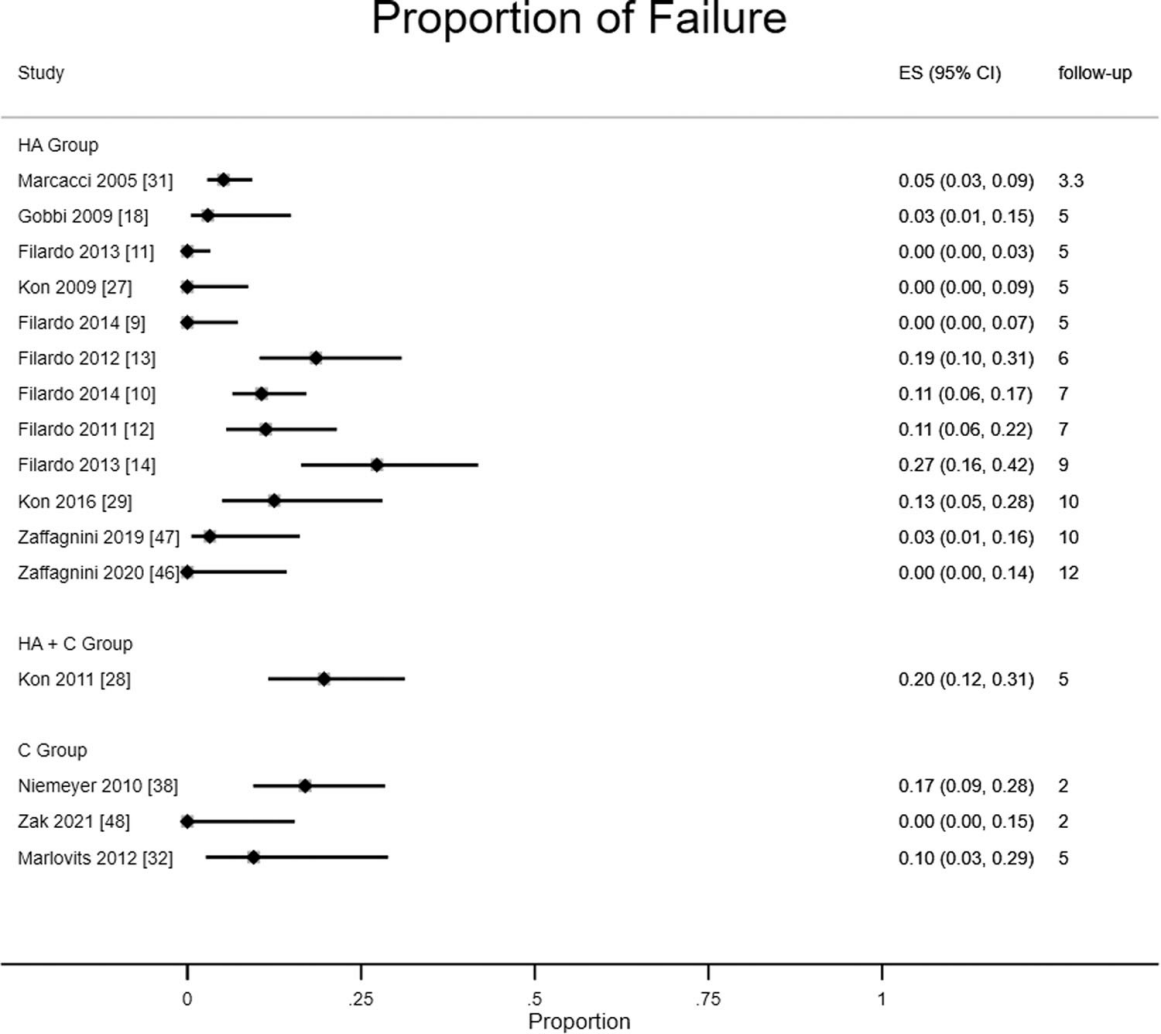
Proportional meta‐analysis of failure rates between HA and C subgroups. C, collagen; HA, hyaluronic acid.

In the HA group, failure rates ranged between 0% and 42%; in the C group, failure rates ranged from 0% and 29%. The study investigating both HA and C groups showed a range of failure proportions between 12% and 31%. Overall, the CIs of failure rate overlapped within and between groups, even considering the different follow‐ups (from 2 to 12 years), meaning that failure rates are similar in the HA group and in the C group over time. Figure 6 shows the failure rates of CIs within and between groups.

## DISCUSSION

The main finding of this systematic review and meta‐analysis is that both scaffolds are suitable to support MACT, as they provided satisfactory medium‐ and long‐term results. The two patients' subgroups, homogeneous in grade, aetiology, and location of the treated defects, showed a significant improvement in all functional scores included in the analysis at 2, 5 and 10 years of follow‐up, when relative data were available. In fact, data on the scores analyzed were not always available at the various follow‐ups considered, where no studies on patients treated with C‐based scaffolds reported follow‐ups longer than 5 years. Therefore, based on the existing literature, it is possible to state that the long‐term outcome (10 years) is confirmed only for MACT performed with an HA‐based scaffold. EQ‐VAS, available only in patients treated with HA‐based scaffold, showed an improvement at 2‐, 5‐ and 10‐year follow‐ups.

Of note, a higher percentage of patients had undergone previous cartilage repair procedures in the HA subgroup than in the C subgroup. Although previous surgery has been reported to be associated with a worse outcome of MACT [36, 49], this does not seem to have a relevant impact on the clinical effectiveness of MACT in patients belonging to the HA subgroup. Conversely, multiple lesions were more common in the C subgroup. However, despite differences in the clinical history of patient cohorts, the procedures were successful in 85%–90% of the treated patients, with a similar failure rate observed for both scaffolds.

Among the reported studies, the first ones in the early 2000s were conducted using HA‐based scaffolds, and only 10 years later, C‐based scaffolds were introduced. This also explains the lack of sufficient data at longer follow‐ups for the latter scaffold. Based on the studies included in this systematic review and meta‐analysis, HA‐based scaffolds were predominantly used in Italy, whereas C‐based scaffolds were utilized in Austria, Mexico, Italy, and Germany. The limited geographic distribution of MACT is due to regulatory hurdles as well as the high costs. Indeed, MACT products must be manufactured in compliance with Good Manufacturing Practices to ensure safety, consistency, and quality. The complexity and cost of manufacturing significantly impact the accessibility and affordability of MACT procedures, albeit the good clinical outcomes observed in the mid‐ and long‐term should promote the diffusion of this therapy.

Comparing the long‐term results of MACT with other surgical techniques for treating articular cartilage injuries, such as the Osteoarticular Autograft Transfer System (OATS), microfractures, and allografts, reveals distinct differences. While MACT outcomes remain stable over time, clinical outcomes for microfractures tend to deteriorate [42]. OATS is generally utilized for smaller injuries because of constraints related to donor site morbidity [23], whereas allografts are not universally available due to regulatory restrictions or tissue unavailability [19].

MACT is a well‐established treatment option for isolated cartilage defects of the knee, providing satisfactory outcomes. However, cases of treatment failure with the need for surgical re‐intervention are reported. We descriptively showed that failure rate CIs overlapped within and between groups, showing no differences between treatments, thus confirming previous findings [39]. On the other hand, a greater reduction in failures has been observed when HA membranes were used compared to C scaffolds at mid‐term follow‐up [35].

However, heterogeneity among studies and between groups might be present: among the 16 out of 19 studies that reported failure rate data, rates occurred in a range between 2 and 10 years, with 7 of them above 5 years of follow‐up. Furthermore, failure includes different types of surgeries, such as cartilage repair procedures, debridement and arthroplasty, leading to differences in outcome reporting between studies. The need for revision surgery represents a well‐established parameter that has been used by many authors in similar studies that evaluate risk factors for failure of various therapies. In the case of MACT, its ability to postpone or avoid more invasive treatments is a key point not only from the individual patient's perspective but also from a broader perspective, since degenerative changes in cartilage are associated with osteoarthritis, which in turn is associated with enormous social costs. The low failure rate identified in the studies included in this systematic review suggests that MACT can be seen as a valid approach to counteract further knee degeneration, or at least to provide a long‐lasting clinical benefit.

The fact that the patients included in this study are young and demonstrated such a low failure rate is reassuring, as most of these patients did not require knee replacement surgery in some cases up to 10 years after the MACT treatment. Treating young patients with prostheses often limits their quality of life and predisposes them to future reoperations for implant substitution [40]. Therefore, promoting regenerative medicine techniques like MACT is crucial, despite their complexity and high cost. Indeed, these techniques can significantly delay, if not entirely prevent, the need for total knee replacements, thereby preserving the patient's quality of life for many years. Although MACT was initially indicated for the treatment of isolated cartilage defects only, it was later used for treating diffuse cartilage lesions, too, with overall satisfactory results [5]. However, the current chondrocyte expansion techniques seem not to be adequate for the treatment of chondral lesions within an OA context, and new findings envision the need for improvements [6].

This study presents some limitations. First, a significant number of studies included in this review are from a limited geographical distribution. In fact, the HA subgroup consists largely of Italian studies. This aspect can potentially introduce biases from research teams, clinical approaches, or patient selection, thus limiting the generalizability of the results. Nevertheless, only two studies, one in the HA subgroup [17] and one in the C subgroup [32], were found to be sponsored by the medical device producer. Consequently, overall, this cannot be seen as a relevant bias. Second, patients documented at the follow‐up in one study may, in some cases, be re‐evaluated in other studies, leading to a partial overlap of patient cohorts between different studies. This can potentially skew the results by inflating the perceived effectiveness of the intervention and underestimating complications, as patients may be counted multiple times in the analysis. Unfortunately, it is impossible to ascertain the exact number of participants being evaluated in more than one study. Third, failure rates were reported inconsistently across studies, with vague definitions of clinical failure (return of symptoms) and structural failure (e.g., graft non‐integration), along with detailed timing for re‐operations [8].

Fourth, the presence of previous and concurrent surgical treatments may have influenced the clinical outcomes of the treated patients. However, this aspect can only be discussed since the data cannot be adjusted to account for it, as the authors did not provide data subgrouped by these features, and the reported scores at each follow‐up are aggregated.

## CONCLUSION

MACT with HA‐ or C‐based scaffolds represents an effective tool of regenerative medicine exploitable for the treatment of cartilage lesions, able to ensure a clinical improvement in the mid‐term for both scaffolds and in the long term for the HA‐based scaffold, and thus suitable to postpone the total knee replacement in young patients.

## AUTHOR CONTRIBUTIONS

**Alessandra Colombini**: Conceptualization; methodology; formal analysis; data curation; writing—original draft preparation; supervision. **Vincenzo Raffo**: Methodology, writing—original draft preparation. **Silvia Gianola**: Methodology; formal analysis; data curation; writing—review and editing. **Greta Castellini**: Methodology; formal analysis; data curation; writing—review and editing. **Silvia Lopa**: Methodology; formal analysis; data curation; writing—review and editing. **Giuseppe Filardo**: Writing—review and editing. **Matteo Moretti**: Writing—review and editing; supervision. **Laura de Girolamo**: Writing—review and editing, Supervision. All authors have read and agreed to the published version of the manuscript.

## CONFLICT OF INTEREST STATEMENT

The authors declare no conflicts of interest.

## ETHICS STATEMENT

Not applicable.

## Supporting information

Supporting information.

## Data Availability

The data sets presented in this study are stored in a data repository at the following link https://osf.io/67akd/?view_only=63a46235ccba48639d42fa09b68551f3.
