# Supplementary material for: Matrix‐assisted autologous chondrocyte transplantation is effective at mid/long‐term for knee lesions: A systematic review and meta‐analysis
Source: Knee Surg Sports Traumatol Arthrosc. 2024 Dec 3;33(8):2866–79. doi: 10.1002/ksa.12549 (PMC12310092; doi:10.1002/ksa.12549)
Supplement: Supplementary file 1 — Supporting information. [file KSA-33-2866-s001.docx]

Supplementary material 1: full search strategy.

Pubmed ("membrane*" OR "scaffold*") AND ("chondrocyte*" OR "cartilage cells") AND "cartilage lesion" OR "cartilage lesions" OR "chondral lesion" OR "chondral lesions" OR "ostheochondral lesion" OR "ostheochondral lesions" OR "cartilage defect" OR "cartilage defects" OR "chondral defect" OR "chondral defects" OR "ostheochondral defect" OR "ostheochondral defects") AND ("human*" OR "patient*") included in the title and/or abstract; for Scopus ( ( membrane* ) OR ( scaffold* ) ) AND ( ( chondrocyte* ) OR ( "cartilage cells" ) ) AND ( ( cartilag* OR chondral OR ostheochondral ) PRE/1 ( ( lesion* ) OR ( defect* ) ) ) AND ( human* OR patient* ) included in title, abstract and keywords; for Cochrane ( ( ( membrane* ) OR ( scaffold* ) ) AND ( ( chondrocyte* ) OR ( "cartilage cells" ) ) AND ( ( cartilag* OR chondral OR ostheochondral ) NEAR/1 ( ( lesion* ) OR ( defect* ) ) ) AND ( human* OR patient* ) ) included in title, abstract and keywords.

Supplementary material 2: Newcastle - Ottawa quality assessment scale cohort studies modified

Selection

1) Representativeness of patients’ cohort

a) truly representative of the average age in the community (1)

b) somewhat representative of the average age in the community (1)

c) selected group of users eg nurses, volunteers (0)

d) no description of the derivation of the cohort (0)

2) Ascertainment of exposure

a) secure record (eg surgical records) (1)

b) structured interview (1)

c) written self report (0)

d) no description (0)

3) Measurement of IKDC before intervention

a) yes (1)

b) no (0)

Type of outcome assessment

1) Assessment of outcome (IKDC)

a) independent blind assessment (1)

b) record linkage (1)

c) self report (0)

d) no description (0)

2) Adequacy of follow-up time

a) yes (select an adequate follow up period for outcome of interest) (1)

b) no (0)

3) Adequacy of follow up of patients’ cohorts

a) complete follow up - all subjects accounted for (1)

b) subjects lost to follow up unlikely to introduce bias - small number lost - > 80 % follow up (1)

c) follow up rate < 80 % and no description of those lost (0)

d) no statement (0)
